# Study on the regulatory mechanism of NsdA_sr_ on rimocidin biosynthesis in *Streptomyces rimosus* M527

**DOI:** 10.1186/s12934-025-02784-z

**Published:** 2025-07-10

**Authors:** Yawen Xie, Yujie Jiang, Yongyong Zhang, Andreas Bechthold, Xiaoping Yu, Zheng Ma

**Affiliations:** 1 Key Laboratory of Microbiological Metrology, Measurement & Bio-Product Quality Security, State Administration for Market Regulation, College of Life Sciences, China Jiliang University, Xueyuan Street, Xiasha Higher Education District, Hangzhou, 310018 Zhejiang Province P.R. China; 2 Institute for Pharmaceutical Sciences, Pharmaceutical Biology and Biotechnology, University of Freiburg, 79104 Freiburg, Germany

**Keywords:** *Streptomyces rimosus*, NsdA_sr_, ChIP-Seq, Precursor supply, GUS

## Abstract

**Background:**

We previously identified a regulator NsdA_sr_, which negatively regulated rimocidin biosynthesis in *Streptomyces rimosus* M527. However, the exact regulatory mechanism of NsdA_sr_ on rimocidin production remains unknown.

**Results:**

In this study, firstly, transcriptomic data demonstrated that the differentially expressed genes resulting from the over-expression of *nsdA*_*sr*_ were primarily associated with several key metabolic pathways, including glycolysis, oxidative phosphorylation, and ribosome-related genes, all of which were downregulated. This directly impacted the concentrations of CoA and NADH, as confirmed by concentration measurement assays. Subsequently, the results of the ChIP-seq experiments revealed that NsdA_sr_ directly binds to 49 target genes. Notably, these include *RS18275* and *RS18290* (both involved in fatty acid degradation) as well as *rpoB* (related to DNA transcription). The validity of the ChIP-seq assay for these three genes was further supported by in vitro electrophoretic mobility shift assays. Regarding *RS18275* and *RS18290*, the results revealed that the binding of NsdA_sr_ to these elements led to the downregulation of gene expression. This, in turn, resulted in a decrease in the levels of butyryl-CoA and malonyl-CoA, which are known precursors for rimocidin biosynthesis. Consequently, this negatively impacted on the biosynthesis of rimocidin. In the case of *rpoB*, the results indicated that NsdA_sr_ binding led to a downregulation of overall protein levels. This was determined by enzymatic activity of report gene GUS and Western blot assay. Consequently, this resulted in a decrease in rimocidin yield.

**Conclusion:**

This study reveals NsdA_sr_’s dual role in limiting rimocidin production by suppressing metabolic precursors and modulating protein expression. Integrated transcriptomic and ChIP-seq analyses provide critical insights into its regulatory mechanisms.

**Supplementary Information:**

The online version contains supplementary material available at 10.1186/s12934-025-02784-z.

## Background

Actinomycetes, particularly streptomycetes, are renowned for their prodigious capacity to produce a diverse array of bioactive secondary metabolites. Many of these metabolites have been clinically harnessed as potent antibacterial, antifungal, antiparasitic, anticancer, and immunosuppressive agents [1–3]. Their applications extend beyond medicine, playing a crucial role in animal husbandry and crop protection [4–8]. In *Streptomyces*, secondary metabolism is under tight control of intricate transcriptional regulatory networks. These sophisticated systems integrate system-wide regulators, internal signal transduction mechanisms, external environmental inputs, and their coordinated executive output cascades [9–12]. Among the regulatory genes involved, *nsdA* has garnered significant attention. Acting as a differentiation suppressor, *nsdA* was first reported in the model actinomycete *Streptomyces coelicolor* A3(2) [13]. The loss of *nsdA* function in *S. coelicolor* triggers coordinated changes: morphological differentiation is accelerated while antibiotic biosynthesis (actinorhodin [Act], methylenomycin, and calcium-dependent antibiotic [CDA]) is derepressed, leading to significantly increased yields. Further research has revealed that *nsdA* similarly exerts negative regulation on morphological differentiation and antibiotic biosynthesis in other streptomycetes. For instance, in *Streptomyces bingchengensis* [14] and *Streptomyces lydicus* A02 [15], disrupting *nsdA* results in higher yields of secondary metabolites, such as natamycin, nanchangmycin, and milbemycin A4. Moreover, compared to their wild-type (WT) strains, these strains with *nsdA* disruption exhibit enhanced pigment and spore formation.

Rimocidin is a glycosylated polyketide belonging to the tetraene macrolide family. Like other macrolides, rimocidin is synthesized in *Streptomyces* species by type I modular polyketide synthases [16]. The extensive antifungal properties of rimocidin against plant pathogens highlight its agricultural application potential while simultaneously making it a compelling research focus [17–19]. *Streptomyces rimosus* M527 is a well-known producer of rimocidin [20] in which a gene transfer system was established [21]. In addition, the production of rimocidin in this strain has been significantly enhanced through ribosome engineering [22] and by adding fungal elicitors to the fermentation medium [23]. However, compared to our comprehensive understanding of its biosynthetic pathway [16, 24, 25], our knowledge of the regulatory mechanisms that control rimocidin biosynthesis and morphological differentiation is still relatively limited. Recent studies have shown that elucidating and manipulating regulatory networks is an effective strategy for further improving the production of various industrial compounds [26, 27].

The gene *nsdA*_*sr*_ was successfully cloned based on the genome sequence of *S. rimosus* M527. It was identified as encoding a protein that exhibits high sequence similarity to NsdA from *S. coelicolor*. Our previous research has demonstrated that NsdA_sr_ functions as a negative regulator of rimocidin biosynthesis, specifically by repressing the transcription of the *rim* genes within the rimocidin biosynthetic gene cluster [28]. The Δ*nsdA*_*sr*_ mutant exhibited increased rimocidin production and accelerated morphological differentiation compared to the WT strain. In contrast, *nsdA*_*sr*_ over-expression resulted in decreased rimocidin production and impaired morphological differentiation. However, the exact molecular mechanism by which NsdA_sr_ affects rimocidin biosynthesis remains unknown.

In this study, we conducted a comparative transcriptomic analysis (*S. rimosus* M527 vs. *S. rimosus* M527-NA_sr_) and identified differentially expressed genes associated with rimocidin biosynthesis. Subsequently, we investigated the molecular mechanism by which NsdA_sr_ regulates rimocidin biosynthesis using ChIP-seq experiments and electrophoretic mobility shift assays (EMSAs).

## Results

### Analysis of differentially expressed genes based on transcriptomic data

To elucidate the differentially expressed genes between *S. rimosus* M527 and M527-NA_sr_, RNA-seq transcriptional analysis between WT strain M527 and M527-NA_sr_ was analyzed at 12 h, 24 h, and 36 h. Construction and sequencing of cDNA library were carried out using the isolated RNA. To ensure the accuracy of the quantitative analysis, low-quality reads, adapter sequences, and reads with a high proportion of unknown bases were removed before proceeding with downstream analysis. Transcriptome sequencing yielded a comprehensive genome map comprising 73,756,859 sequence reads, all of which were precisely aligned to the reference genome of *S. rimosus*. More than 87% of the reads were mapped to the *S. rimosus* genome, indicating reliable sample quality and high consistency with the reference genome (Supplementary file: Table S1). Additionally, the average unique mapped reads ratio reached 87%, demonstrating that the sequencing data was of high quality and that the genome annotation was sufficiently complete for in-depth studies of gene expression and function.

**Fig. 1:**
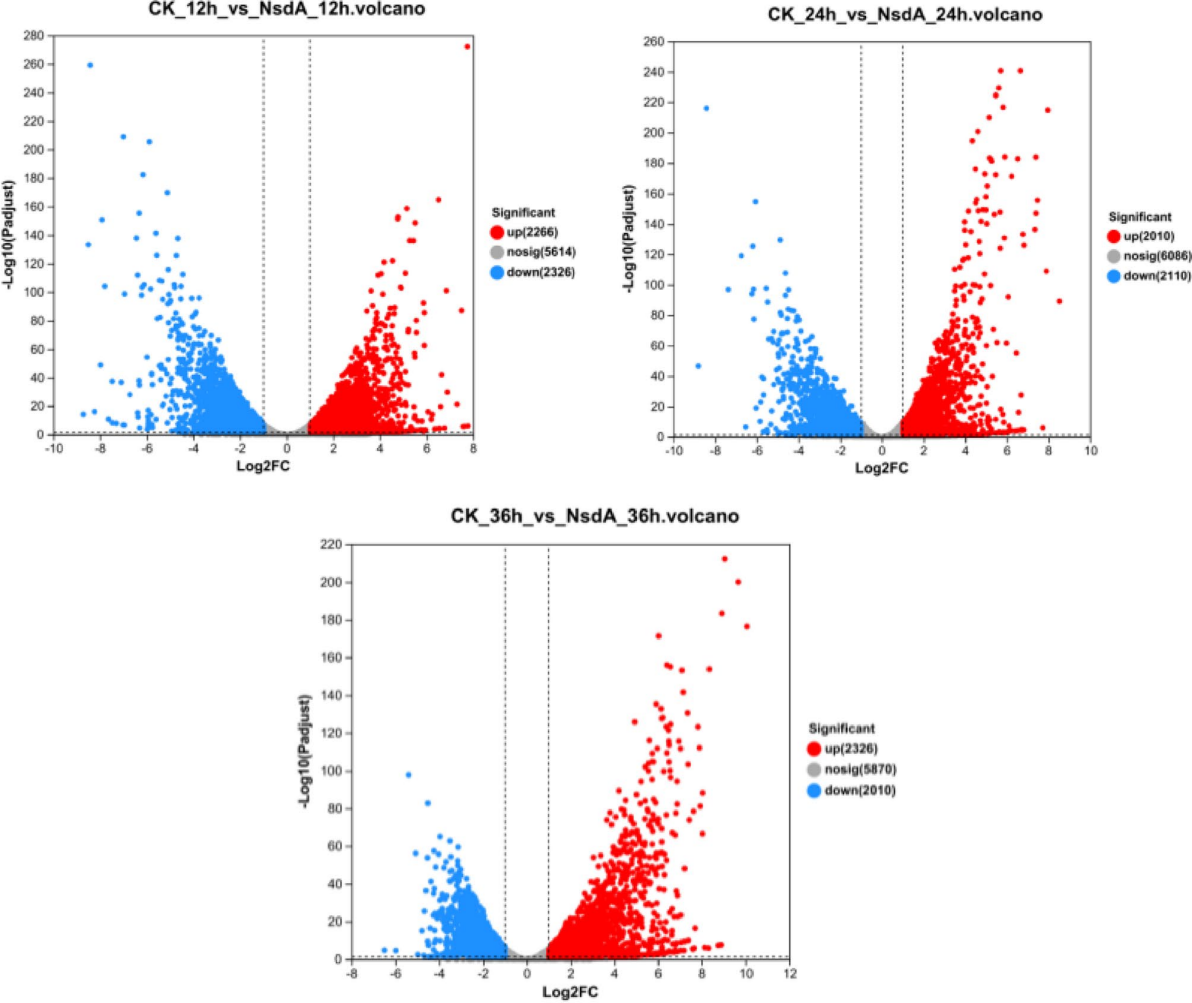
Volcano-plots of differentially expressed genes. Each dot in the graph represents a gene, and the red and blue dots correspond to genes that are significantly upregulated and downregulated, respectively, while the gray dots are not significant

RNA-seq analysis unveiled a substantial number of differentially expressed genes between the WT strain M527 and M527-NA_sr_, as vividly depicted in the volcano plots (Fig. 1). Specifically, 2,266 genes were up-regulated and 2,326 genes downregulated in M527-NA_sr_ at 12 h, 2,010 genes were up-regulated and 2,110 genes downregulated at 24 h, and 2,326 genes were up-regulated and 2,010 genes downregulated at 36 h.

By comparing transcriptomic data across different time points, gene expression differences between M527-NA_sr_ and the WT strain M527 were analyzed. Several differentially expressed genes involved in amino acid metabolism were significantly downregulated in M527-NA_sr_ compared to M527, including genes from the aspartate, glutamate, and alanine metabolism pathways, as well as the serine, glycine, and threonine metabolism pathways. Additionally, transcriptional levels of differentially expressed genes in ribosome biosynthesis and carbohydrate metabolism pathways, such as pyruvate metabolism, butanoate metabolism, and propanoate metabolism, were decreased. Moreover, genes involved in signal transduction, quorum sensing, and transport pathways were also downregulated in M527-NA_sr_ (Supplementary file: Table S2).

A detailed transcriptomic analysis revealed significant downregulation of ribosome-related genes in M527-NA_sr_ (Table 1). The consistent decrease in transcriptional levels of ribosome-related genes suggests that the over-expression of *nsdA*_*sr*_ caused a decline in translation efficiency. Furthermore, genes involved in oxidative phosphorylation and glycolysis/ gluconeogenesis were also downregulated. Oxidative phosphorylation is a major energy acquisition pathway in bacteria. Through the electron transport chain, bacteria transfer electrons from NADH and FADH_2_ to oxygen molecules, driving ATP synthase to produce ATP. These ATP molecules provide energy for various cellular activities. Secondary metabolite biosynthesis and cell growth typically rely on a sufficient ATP supply, whereas energy shortages shift cellular priorities toward primary metabolism. Similarly, glycolysis provides key intermediates such as pyruvate and acetyl-CoA, which serve as essential building blocks for secondary metabolites in *Streptomyces*. Reduced expression of glycolytic genes may limit the flux of these intermediates, thereby constraining secondary metabolite biosynthesis.

**Table 1:** Analysis of KEGG metabolic pathways

Gene_ID	Gene name	Gene description^*^	NA vs. CK		
			12 h	24 h	36 h
Ribosome biosynthetic					
EMY36_RS05365	*rplP*	50 S ribosomal protein L16	/	-1.96	-2.98
EMY36_RS05360	*EMY36_RS05360*	50 S ribosomal protein L29	-1.22	-1.99	-2.84
EMY36_RS05350	*rplN*	50 S ribosomal protein L14	-1.03	-1.8	-2.2
EMY36_RS05380	*rpsS*	30 S ribosomal protein S19	/	-1.04	-1.85
EMY36_RS05370	*rpsC*	30 S ribosomal protein S3	/	-1.9	-2.23
ABC transporters					
EMY36_RS20510	*ngcE*	N-acetylglucosamine/diacetylchitobiose ABC transporter substrate-binding protein	-4.75	-3.28	-3.08
EMY36_RS20515	*EMY36_RS20515*	sugar ABC transporter permease	-4.23	-2.78	-2.72
EMY36_RS22945	*EMY36_RS22945*	sugar ABC transporter permease	-3.67	-2.15	-1.56
EMY36_RS19035	*EMY36_RS19035*	glutamate ABC transporter substrate-binding protein	-3.77	-2.48	-3.15
EMY36_RS22940	*EMY36_RS22940*	carbohydrate ABC transporter permease	-4.13	-1.91	-1.18
EMY36_RS38560	*EMY36_RS38560*	dipeptide ABC transporter ATP-binding protein	-4.1	-1.24	-1.38
EMY36_RS32600	*EMY36_RS32600*	ABC transporter permease	-6.12	-1.23	-1.04
Oxidative phosphorylation					
EMY36_RS05750	*nuoL*	NADH-quinone oxidoreductase subunit L	-1.23	/	-2
EMY36_RS05785	*nuoE*	NADH-quinone oxidoreductase subunit NuoE	-1.08	/	-1.75
EMY36_RS05765	*nuoI*	NADH-quinone oxidoreductase subunit NuoI	-1.15	/	-1.58
EMY36_RS05740	*nuoN*	NADH-quinone oxidoreductase subunit NuoN	-1.41	/	-1.91
EMY36_RS05780	*nuoF*	NADH-quinone oxidoreductase subunit NuoF	-1.03	/	-1.3
EMY36_RS05770	*nuoH*	NADH-quinone oxidoreductase subunit NuoH	-1.37	/	-1.74
EMY36_RS31645	*atpE*	ATP synthase F0 subunit C	-2.39	/	-1.46
EMY36_RS31620	*atpD*	F0F1 ATP synthase subunit beta	-2.17	/	-1.4
Glycolysis / Gluconeogenesis					
EMY36_RS26150	*EMY36_RS26150*	Zn-dependent alcohol dehydrogenase	-2.08	-2.31	/
EMY36_RS07345	*EMY36_RS07345*	acetate--CoA ligase family protein	-1.67	-2.59	/
EMY36_RS35440	*EMY36_RS35440*	aldehyde dehydrogenase family protein	-3.23	-1.81	-1.66
EMY36_RS35085	*acs*	acetate--CoA ligase	-1.36	-1.36	-1.42
EMY36_RS11880	*EMY36_RS11880*	aldehyde dehydrogenase	-1.56	-1.90	/
EMY36_RS26145	*EMY36_RS26145*	aldehyde dehydrogenase family protein	-2.38	-2.67	/

^*^The putative functions of genes were predicted based on most similar product *via* comparison of reference genome of *Streptomyces rimosus subsp. rimosus* ATCC 10,970 (GCF_000331185.2)

### Analysis of rimocidin precursor biosynthesis genes

**Fig. 2:**
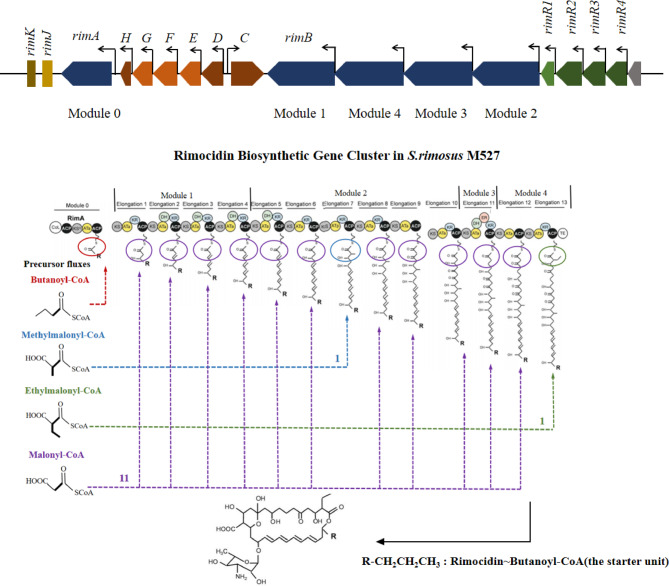
Gene organization of *rim* gene cluster in the genome of *Streptomyces rimosus* M527 and rimocidin biosynthetic pathway, where acetyl-CoA, methylmalonyl-CoA, ethylmalonyl-CoA, butanoyl-CoA and malonyl-CoA are involved in. Proposed model for rimocidin and CE-108 biosynthesis in *S. diastaticus* var. 108 (Seco et al., 2004), R: -CH_3_ (CE-108, acetyl-CoA as the starter unit); R: -CH_2_CH_2_CH_3_ (rimocidin, butanoyl-CoA as the starter unit). The functional annotation of genes in *rim* gene cluster is consistent with the previous report [40]

**Fig. 3:**
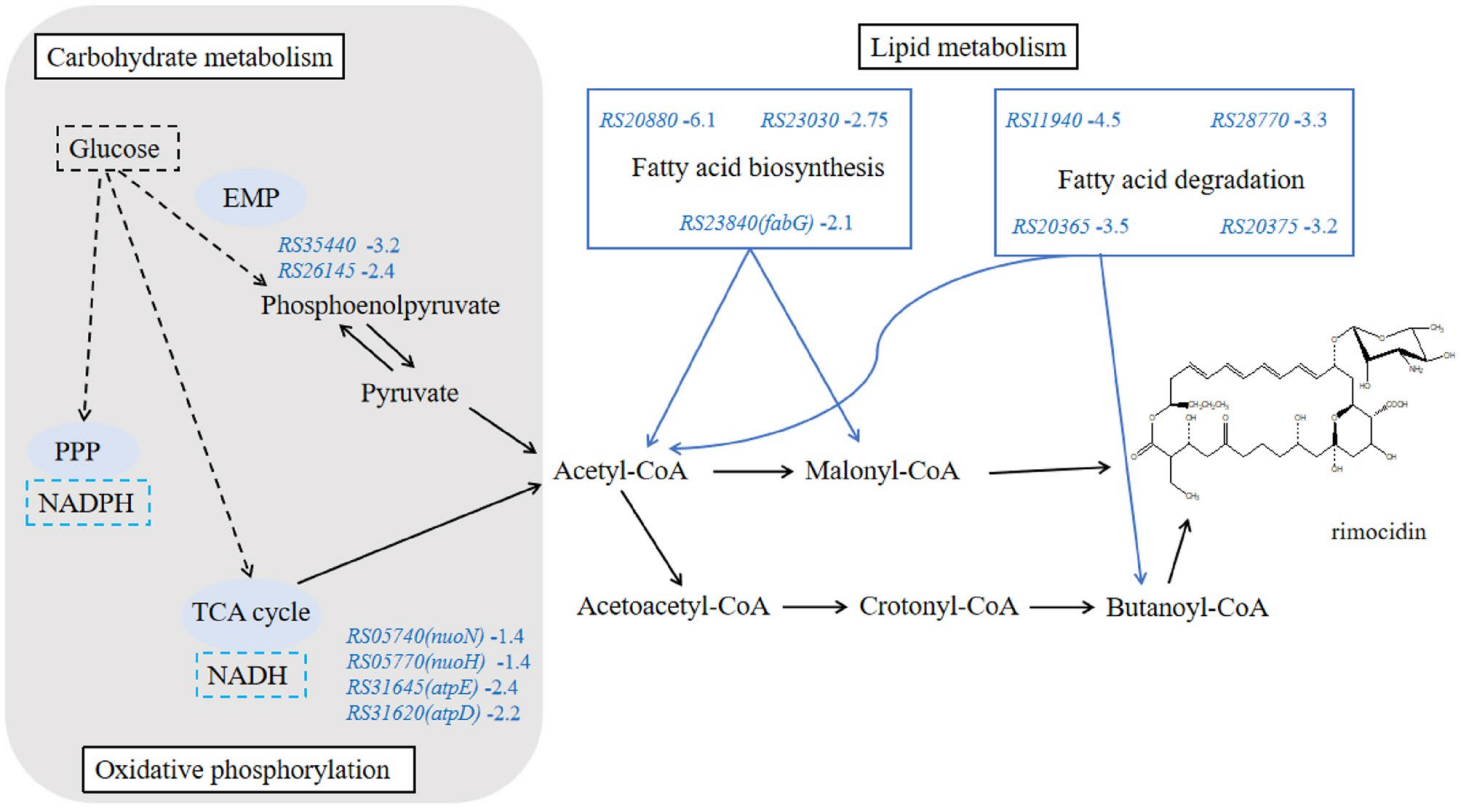
The KEGG analysis of selected DEGs and the resulting schematic overview of primary metabolic changes in *S. rimosus* M527-NA_sr_: the number behind DEG was the log_2_ (fold change) of transcription level, the dashed arrow indicated multi-step reactions, the downregulated pathway was highlighted in blue (*fabG*: 3-oxoacyl-ACP reductase FabG; *RS20365*: enoyl-CoA hydratase; *RS20375*: enoyl-CoA hydratase/isomerase family protein)

The transcription of genes involved in the biosynthesis of rimocidin precursors was analyzed at 12 h, 24 h, and 36 h. Methylmalonyl-CoA, ethylmalonyl-CoA, butanoyl-CoA, and malonyl-CoA were identified as key precursors in rimocidin biosynthesis. The biosynthesis of a single rimocidin molecule requires the utilization of up to 11 malonyl-CoA units (Fig. 2). These precursors or intermediates originate from primary metabolic pathways, including amino acid catabolism, fatty acid degradation, the citric acid cycle, and pyruvate metabolism. The *fabG* gene, which is involved in malonyl-CoA biosynthesis through fatty acid metabolism (Table 2), exhibited a 2.14-fold transcriptional downregulation in M527-NA_sr_ during the first 12 h. In our previous studies, intracellular malonyl-CoA concentrations were increased by over-expressing the *acc*_*sr*_ gene, resulting in a 30% improvement in rimocidin yield, confirming the impact of malonyl-CoA abundance on rimocidin biosynthesis. Thus, the downregulation of *fabG* expression partially inhibits rimocidin biosynthesis. Similarly, mRNA abundance of butanoyl-CoA biosynthesis-related genes (*EMY36_RS20365*, *EMY36_RS20375*) was reduced more than threefold, indicating a diminished precursor supply for rimocidin biosynthesis (Fig. 3). Overall, over-expression of *nsdA*_*sr*_ resulted in insufficient reducing power and acyl-CoA availability, leading to decreased rimocidin biosynthesis.

**Table 2:** Analysis of metabolic pathways involved in precursor synthesis

Gene_ID	Gene name	Most similar product^*^	NA vs. CK		
			12 h	24 h	36 h
Pyruvate metabolism					
EMY36_RS14400	*EMY36_RS14400*	pyruvate dehydrogenase	4.75	5.15	6.2
EMY36_RS31320	*pta*	phosphate acetyltransferase	3.11	2.57	2.37
EMY36_RS13750	*EMY36_RS13750*	NAD(P)-dependent alcohol dehydrogenase	2.17	3.09	3.16
EMY36_RS23030	*EMY36_RS23030*	ATP-grasp domain-containing protein	-2.75	3.61	1.62
Fatty acid biosynthesis/degradation					
EMY36_RS23840	*fabG*	3-oxoacyl-ACP reductase FabG	-2.14	-1.2	-1.12
EMY36_RS20365	*EMY36_RS20365*	enoyl-CoA hydratase	-3.5	-2.93	-2.9
EMY36_RS20375	*EMY36_RS20375*	enoyl-CoA hydratase/isomerase family protein	-3.17	-2.01	-2.55
EMY36_RS28770	*EMY36_RS28770*	crotonase/enoyl-CoA hydratase family protein	-3.25	-2.99	/
EMY36_RS11940	*EMY36_RS11940*	steroid 3-ketoacyl-CoA thiolase	-4.52	-4.81	-2.25
EMY36_RS20880	*EMY36_RS20880*	aldehyde dehydrogenase family protein	/	-6.07	-1.23

^*^The putative functions of genes were predicted based on most similar product *via* comparison of reference genome of *Streptomyces rimosus subsp. rimosus* ATCC 10,970 (GCF_000331185.2)

### Determination of intracellular NADH and NADPH in *S. rimosus* M527

**Fig. 4:**
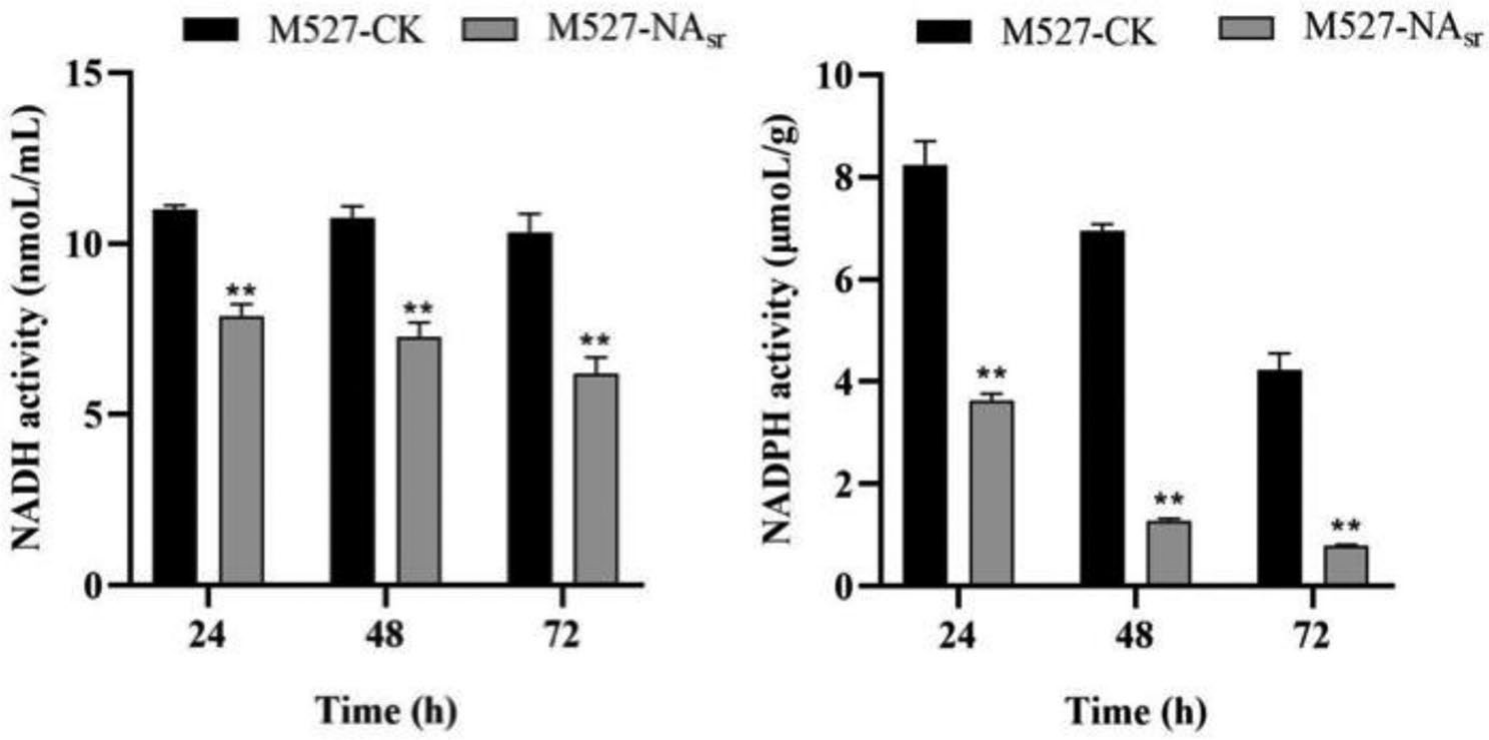
Determination of intracellular NADH and NADPH concentrations in *S. rimosus* M527/M527-NA_sr_. ^**^Indicates highly statistically significant results (*P* value < 0.01)

Based on transcriptomic analysis, we hypothesized that downregulation of the expression level of some genes, including oxidative phosphorylation-related genes, may cause changes in the intracellular concentrations of NADH and NADPH in *S. rimosus* M527. To test this, intracellular concentrations of NADH and NADPH were measured in *S. rimosus* M527-NA_sr_ and *S. rimosus* M527. In M527-NA_sr_, intracellular NADH levels were 28.4–40.1% lower than those in the WT strain, while intracellular NADPH levels were 56–81.6% lower (Fig. 4). These results indicated that *nsdA*_*sr*_ over-expression resulted in lower intracellular concentrations of NADH and NADPH.

### Construction of recombinant strain *S. rimosus* M527-NA_his_

NsdA_sr_ has been confirmed to be a negative regulator. This suggests that an increase in the copy number of *nsdA*_*sr*_ has a negative impact on morphological differentiation and rimocidin production [28]. To analyze the specific targets of NsdA_sr_ in *S. rimosus* M527, the *nsdA*_*sr*_ gene was tagged with a His tag and placed under the control of the strong promoter *ermE* in plasmid pIB139 to yield pIB139-*nsdA*_*his*_ (Supplementary file: Figure S1 and Figure S2). The plasmid was introduced and integrated into the chromosome of *S. rimosus* M527 by conjugation, generating the recombinant strain M527-NA_his_, which exhibited resistance to apramycin (Supplementary file: Figure S3). Furthermore, the successful integration of the pIB139-*nsdA*_*his*_ was confirmed through PCR analysis (Supplementary file: Figure S4).

### Over-expression of *nsdA*_*his*_ inhibits rimocidcin production

**Fig. 5:**
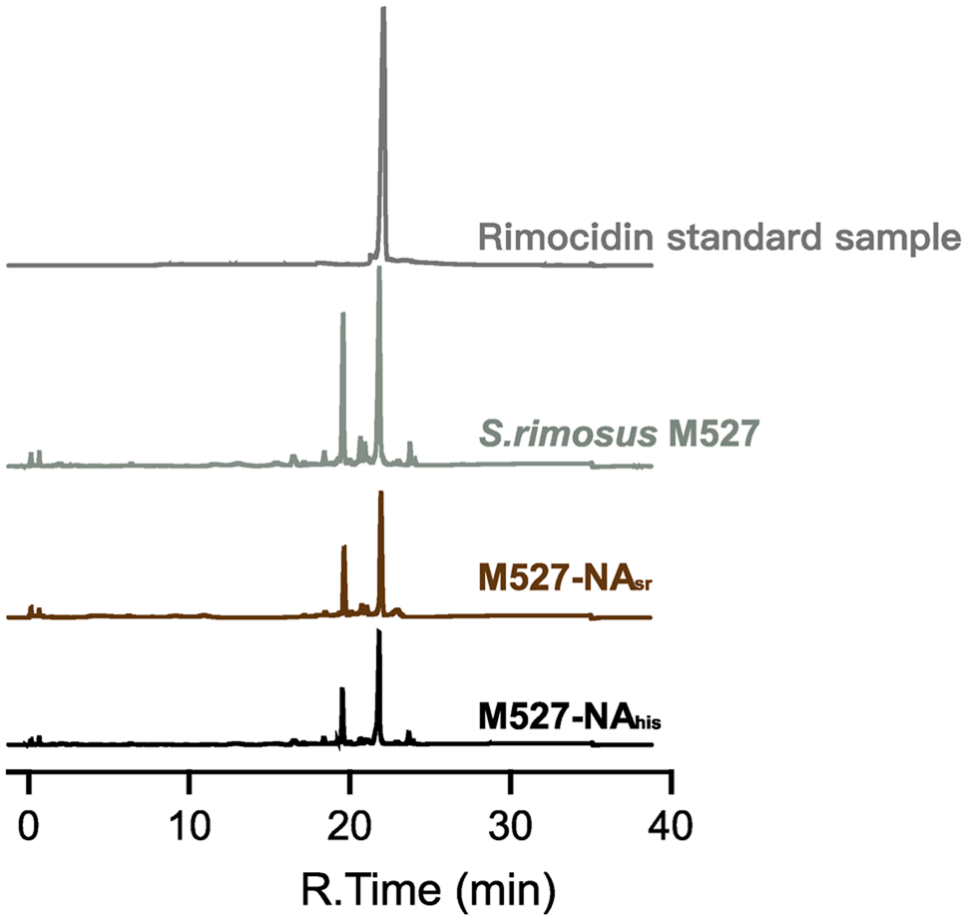
HPLC analysis of rimocidin production from fermentation extracts of the WT strain *S. rimosus* M527, M527-NA_sr_, and M527-NA_his_

**Fig. 6:**
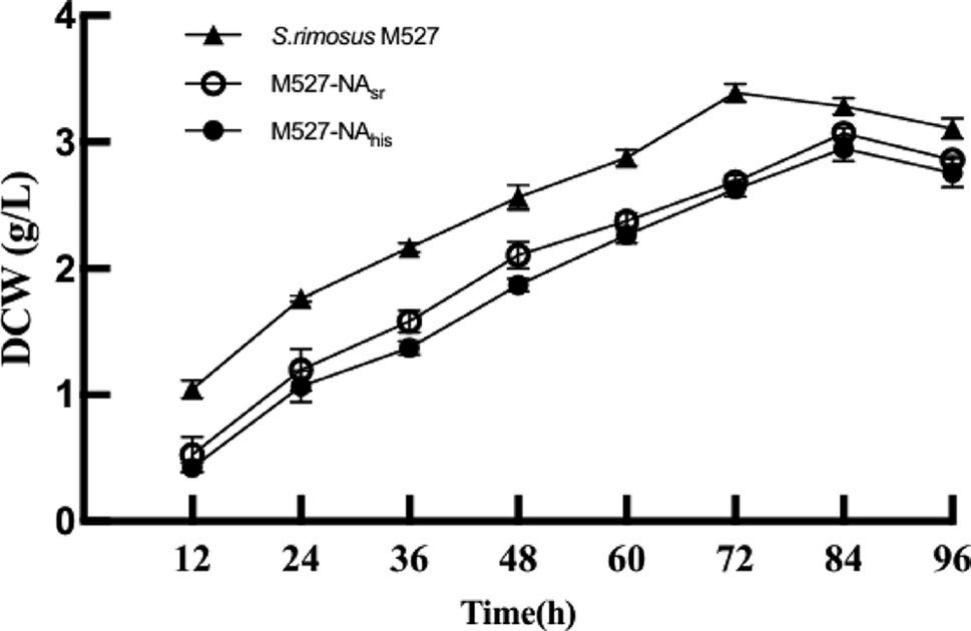
Analysis and comparison of dry cell weight (DCW) in WT strain *S. rimosus* M527, recombinant strain M527-NA_sr_, and M527‐NA_his_ in shake‐flask culture experiment. Error bars indicate SD of samples performed in triplicate

The His tag, a widely used commercial antibody with high specificity and efficiency, facilitates immunoprecipitation and helps isolate NsdA_sr_ binding sites using anti-His antibody-based immunoprecipitation. To assess the impact of NsdA_his_ on rimocidin production and cell growth, the recombinant strain M527-NA_his_ and WT strain M527 were both fermented at shake-flask level. As shown in Fig. 5, rimocidin production in M527-NA_his_ was significantly reduced. After 72 h, the maximum rimocidin yield in M527-NA_his_ was 93.5 mg/L, representing a 56.2% decrease compared to the 213.6 mg/L yield in the WT strain. Notably, rimocidin production in M527-NA_his_ was similar to that in M527-NA_sr_, indicating that the His tag did not affect *nsdA* gene expression. Additionally, over-expression of *nsdA*_*his*_ exhibited the same negative effects on the cell growth (Fig. 6). Similarly, there was no significant difference between M527-NA_his_ and M527-NA_sr_ in terms of cell growth.

**Fig. 7:**
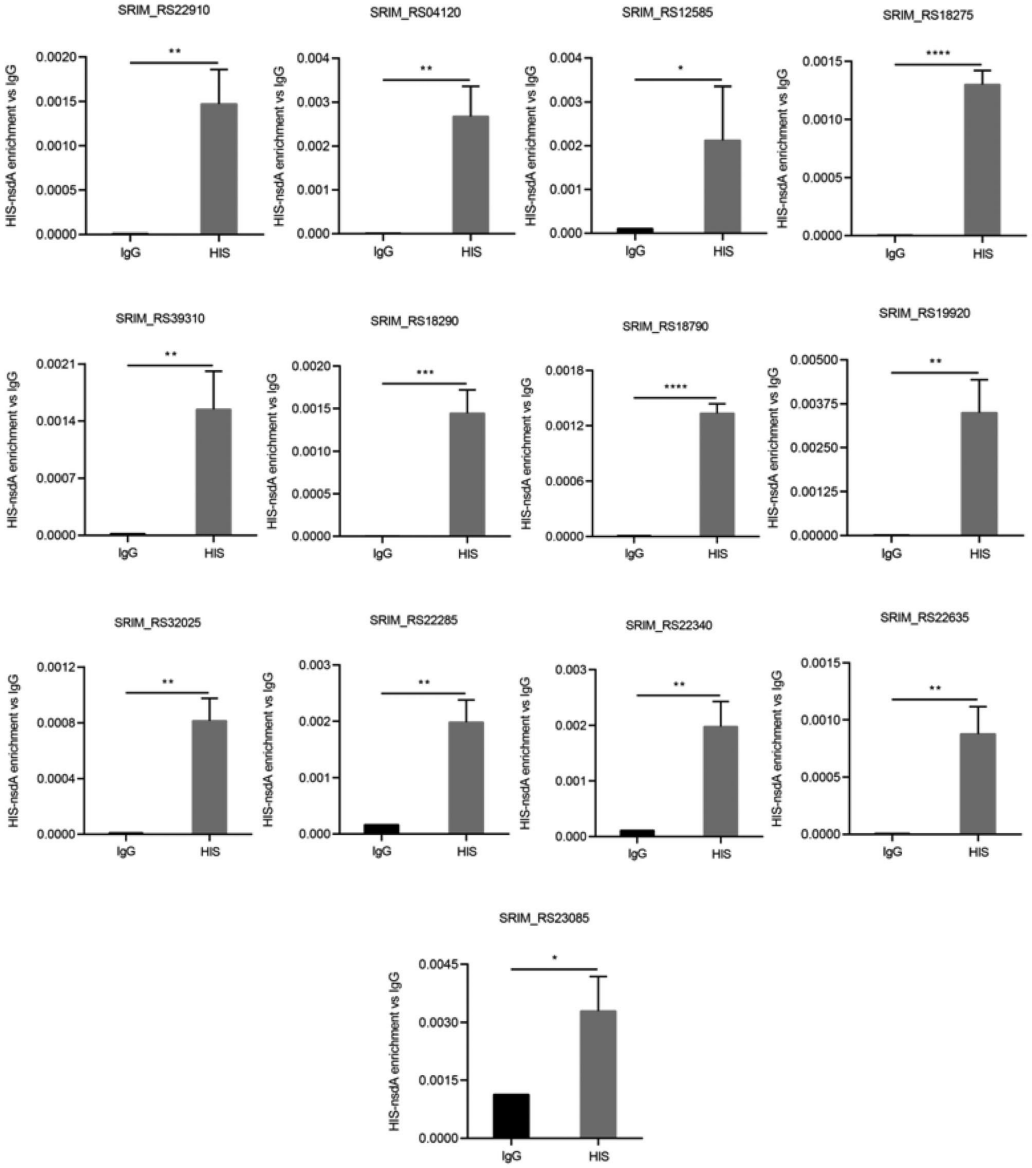
Verification of selected target genes of M527-NA_his_ by ChIP-qPCR. Statistical significance: *0.01 <*P* < 0.05, **P < 0.01

**Fig. 8:**
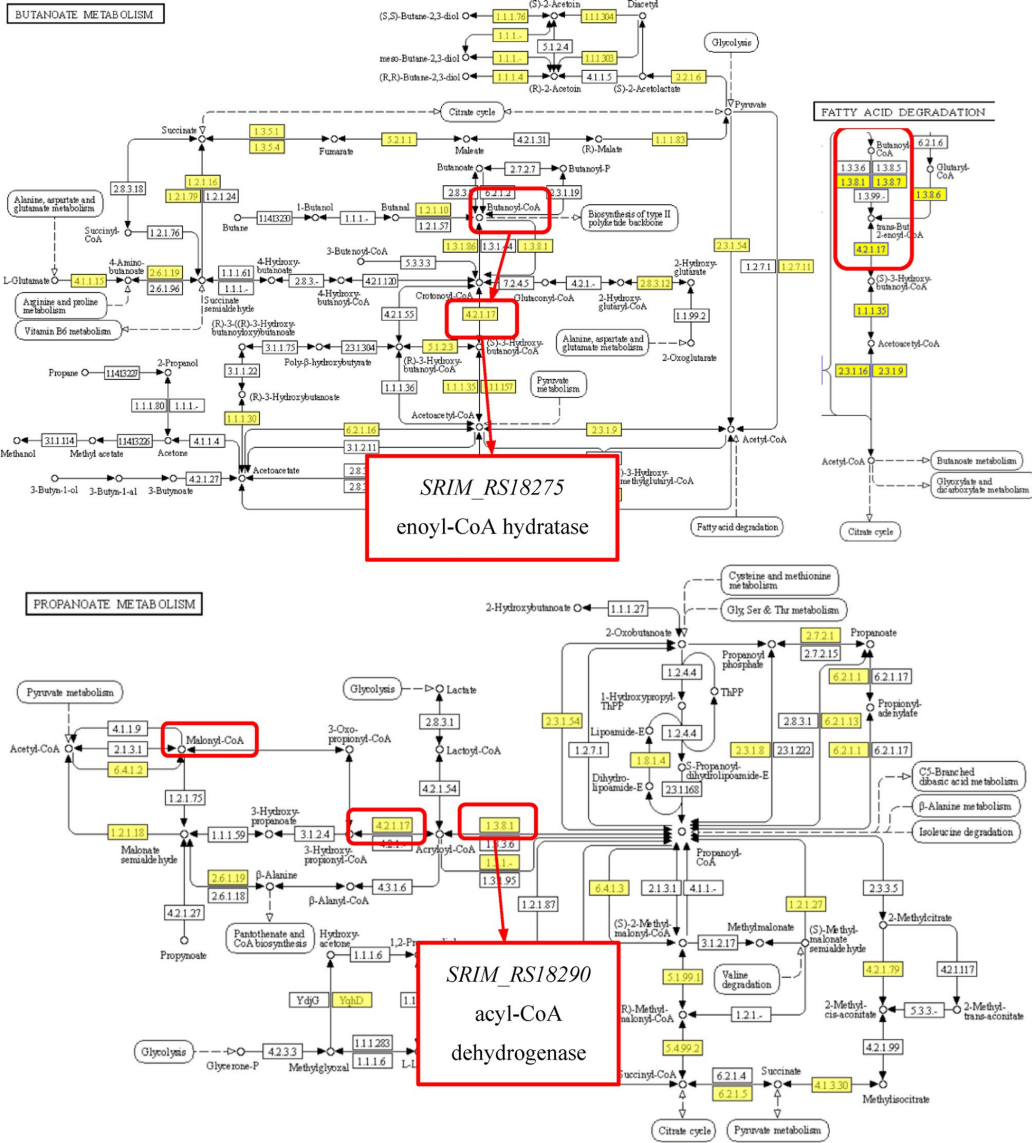
Butanoate, propanoate metabolism and fatty acid degradation pathway in *S. rimosus* M527-NA_his_

**Fig. 9:**
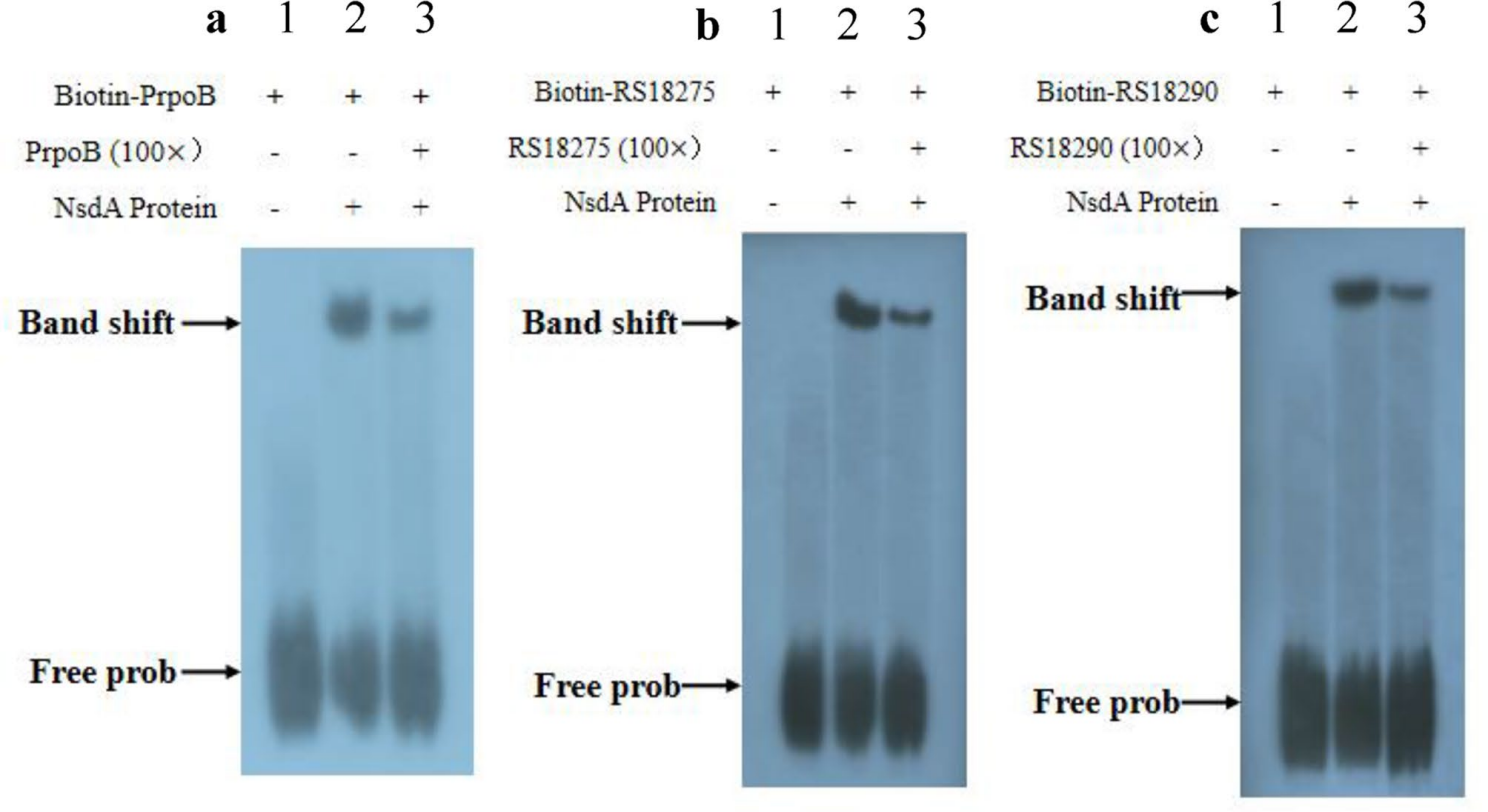
In vitro EMSA of NsdA_sr_ binding to the putative promoter regions of genes *rpoB*(**a**), *RS18275*(**b**) and *RS18290*(c). His_6_-NsdA_sr_ protein was incubated with biotinylated promoter probes, with a 100× molar excess of unlabeled competitor DNA added in competition assays. NsdA_sr_ protein binding putative promoter region of genes *rpoB*(**a**), *RS18275*(**b**) and *RS18290*(**c**). The symbols “+” or “−” in the top row indicate the presence or absence of probes and competitors. Lane 1: biotin-labeled DNA probe; lane 2: biotin-labeled DNA probe plus NsdA_sr_ protein; lane 3: a 100-fold excess of unlabeled specific competitor plus NsdA_sr_ protein. DNA-protein binding conditions: 0.04 pmol/μL biotinylated probe with 10 μg NsdA_sr_

**Fig. 10:**
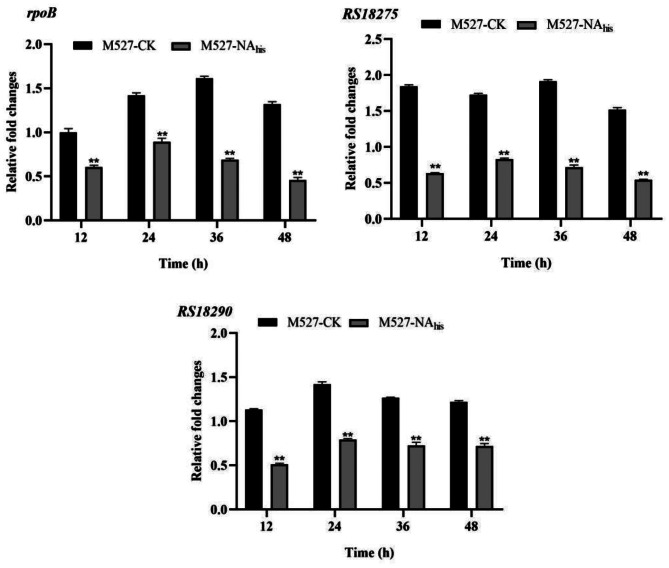
Transcript levels of *rpoB*, *RS18275*, and *RS18290* were compared between WT *S. rimosus* M527 and recombinant strain M527-NA_his_ using quantitative RT-PCR, with error bars representing standard deviation from three biological replicates (**P<0.01)

**Fig. 11:**
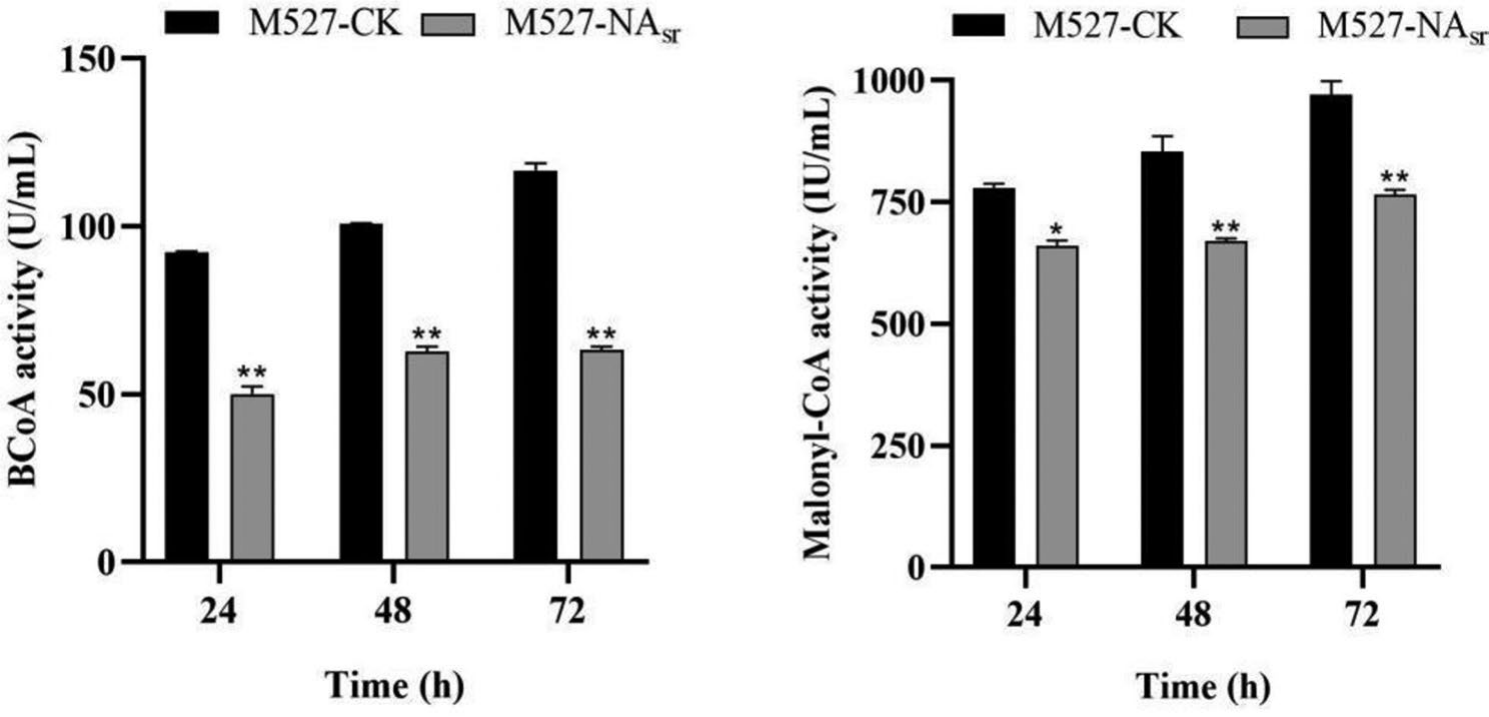
Determination of intracellular butanoyl-CoA and malonyl-CoA in *S. rimosus* M527 and M527-NA_sr_. Data are shown as mean ± SD from three biological replicates. (**P<0.01)

**Fig. 12:**
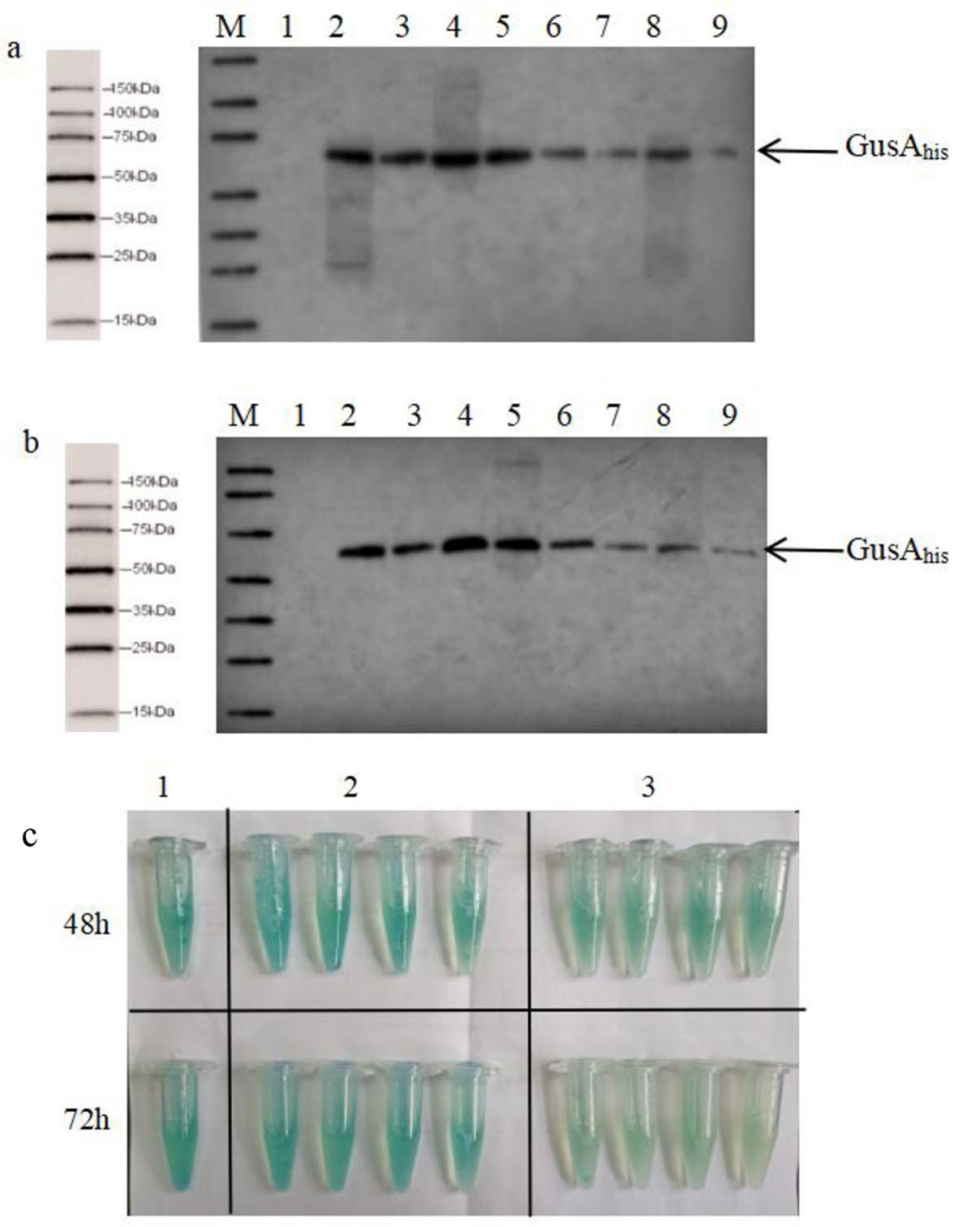
Determination of the GUS activity and total protein content of *S. rimosus* M527-GA_his_ and M527-NGA_his_. a: Western blot detection of M527-GA_his_ and M527-NGA_his_. M: 150 kDa protein Marker. Lane 1, negative control M527-ES; Lane 2-3, M527-GA_his_-1 48 h/72 h protein; Lane 4-5, M527-GA_his_-2 48 h/72 h; Lane 6-7, M527-NGA_his_-1 48 h/72 h; Lane 8-9, M527-NGA_his_-2 48 h/72 h. b: Western blot detection of M527-GA_his_ and M527-NGA_his_. M: 150 kDa protein Marker. Lane 1, negative control M527-ES; Lane 2-3, M527-GA_his_-3 48 h/72 h protein; Lane 4-5, M527-GA_his_-4 48 h/72 h; Lane 6-7, M527-NGA_his_-3 48 h/72 h; Lane 8-9, M527-NGA_his_-4 48 h/72 h. c: GUS activity phenotype detection. 1: M527-ES-48 h/72 h; 2: M527-GA_his_-48 h/72 h; 3: M527-NGA_his_-48 h/72 h

### Analysis of NsdA_sr_ binding to potential genes in vivo using ChIP-qPCR

Based on the genome sequence of *S. rimosus*, gene annotation was performed to identify DNA sequences bound to the NsdA_sr_ protein. Using *P* < 0.05 as the threshold for statistical significance, 49 peaks were identified with enrichment factors ranging from 1.2 to 1.5. Among these, 13 genes with predicted functions potentially related to rimocidin biosynthesis were selected through functional classification (Table 3). According to the predicted function, these genes were involved in the fatty acid degradation pathway (*RS18275* and *RS18290*), glucose metabolism (*pflB*), DNA transcription (*rpoB*), and others.

**Table 3:** Analysis of some peak genes in strain M527-NA_his_ by ChIP-seq

Gene ID	Most similar product^*^	Note
SRIM_RS04120(*pflB*)	formate C-acetyltransferase	glycometabolism
SRIM_RS12585	N-acetyltransferase	acyltransferase activity
SRIM_RS18275	enoyl-CoA hydratase-related protein	fatty acid degradation
SRIM_RS18290	acyl-CoA dehydrogenase family protein	fatty acid degradation
SRIM_RS18790(*rpoB*)	DNA-directed RNA polymerase subunit beta	DNA transcription
SRIM_RS19920(*bldC*)	developmental transcriptional regulator BldC	DNA transcription
SRIM_RS22285	glycosyltransferase family 39 protein	glycosyltransferase activity
SRIM_RS22340	Monooxygenase	FAD binding
SRIM_RS22635	GMC family oxidoreductase	FAD binding
SRIM_RS22910	helix-turn-helix domain-containing protein	DNA binding
SRIM_RS23085	WhiB family transcriptional regulator	DNA transcription
SRIM_RS32025	helix-turn-helix domain-containing protein	DNA binding
SRIM_RS39310	4Fe-4 S dicluster domain-containing protein	iron-sulfur cluster binding

^*^The putative functions of genes were predicted based on most similar product *via* comparison of reference genome of *Streptomyces rimosus subsp. rimosus* ATCC 10,970 (GCF_000331185.2)

To further evaluate the in vivo binding sites of NsdA_sr_, ChIP-qPCR was performed on its predicted target genes (Fig. 7). Data analysis revealed that the M527-NA_his_ strain exhibited significantly higher binding levels at *RS04120* (*pflB*), *RS12585*, *RS18275*, *RS18290*, *RS18790* (*rpoB*), *RS19920* (*bldC*), *RS22285*, *RS22340*, *RS22635*, *RS22910*, *RS32025*, and *RS39310*. The binding difference observed for *RS23085* (0.01 < *P* < 0.05) suggested a potential false positive; thus, this data point was excluded. These results indicate that NsdA_sr_ is capable of binding these sites in vivo. For the predicted NsdA_sr_ binding site at *RS23085*, the ChIP-qPCR signal for NsdA-His was comparable to the IgG control, indicating no specific binding under the tested conditions.

### NsdA_sr_ targets *rpoB*, *RS18275*, and *RS18290*

Among the identified target genes, NsdA_sr_ was found to bind to the promoter region of *rpoB.* This gene encodes the β subunit of RNA polymerase that is pivotal in bacterial transcription. Suppression of *rpoB* expression significantly reduces RNA polymerase activity, leading to decreased transcription efficiency. This inhibition impacts bacterial growth rate, metabolic activity, adaptability to environmental stress, and both central and secondary metabolic pathways collectively control metabolite biosynthesis.

Additionally, *RS18275* and *RS18290* are involved in multiple KEGG metabolic pathways, including butanoate metabolism, propanoate metabolism, and fatty acid degradation (Fig. 8). Alterations in their expression can significantly affect the synthesis of key metabolites, particularly butanoyl-CoA and malonyl-CoA, leading to impaired metabolite production, including a notable reduction in rimocidin biosynthesis.

### Verification of target genes in vitro using EMSA

To further investigate NsdA_sr_ binding, the vector pET28a-*nsdA*_*sr*_ was constructed (Supplementary file: Figure S5) and transformed into *E. coli* BL21 (DE3). The NsdA_sr_ protein was induced with IPTG and purified (Supplementary file: Figure S6). EMSA was conducted using probes containing the predicted binding sequences of three target genes (Fig. 9). The results demonstrated that NsdA_sr_ binds these predicted sites with varying affinities, confirming that these genes are potential direct targets of NsdA_sr_.

### Relative expression levels of NsdA_sr_-regulated target genes

To assess the regulatory impact of NsdA_sr_, a quantitative real-time PCR (qRT-PCR) approach was implemented to measure expression changes in *rpoB*, RS18275 and RS18290 genes. RNA was extracted from independent biological samples of WT strain M527 and M527-NA_his_ strain, which were cultured in PDA liquid medium for 12, 24, 36, and 48 h. qRT-PCR analysis revealed significantly lower expression of these genes in M527-NA_his_ compared to the WT M527 strain (Fig. 10).

### Determination of intracellular malonyl-CoA and butanoyl-CoA in *S. rimosus* M527

Since *RS18275* and *RS18290* are involved in key metabolic pathways, we hypothesized that their decreased transcription could lead to reduced intracellular levels of butanoyl-CoA and malonyl-CoA in *S. rimosus* M527. To test this, the intracellular concentrations of butanoyl-CoA and malonyl-CoA were measured in *S. rimosus* M527-NA_sr_ and *S. rimosus* M527. The results confirmed that over-expression of *nsdA*_*sr*_ reduced the transcription of *RS18275* and *RS18290*, leading to lower intracellular metabolite levels. In *S. rimosus* M527-NA_sr_, butanoyl-CoA levels were 37.85–45.85% lower, and malonyl-CoA levels were 15.37–21.53% lower than those in *S. rimosus* M527 (Fig. 11).

### Construction of recombinant strains *S. rimosus* M527-GA_his_/M527-NGA_his_

To further elucidate the regulatory role of NsdA_sr_ in *rpoB* and its impact on rimocidin biosynthesis, a DNA fragment harboring the *gusA*_*his*_ gene, which encodes β-glucuronidase (GUS), was placed under the control of the strong promoter ermE^*^ in plasmid pIB139, thereby generating pIB139*-gusA*_*his*_ (Supplementary file: Figure S7 and Figure S8). Using pIB139*-gusA*_*his*_ as a template, a gene fragment containing promoter ermE^*^ and *gusA*_*his*_ was cloned and inserted into pIB139-*nsdA*, resulting in the recombinant plasmid pIB139-*nsdA-gusA*_*his*_ (Supplementary file: Figure S9 and Figure S10). The plasmids pIB139-*gusA*_*his*_ and pIB139*-nsdA-gusA*_*his*_ were introduced into *S. rimosus* M527 through intergeneric conjugative transfer from *E. coli* to *Streptomyces*, resulting in the generation of recombinant strains *S. rimosus* M527-GA_his_/M527-NGA_his_. The phenotypic verification and PCR analysis of recombinant strains *S. rimosus* M527-GA_his_/M527-NGA_his_ were also carried out (data not shown).

### GUS reporter assay confirms NsdA_sr_ over-expression downregulates protein expression in *S. rimosus* M527

The *gusA* gene was expressed in *S. rimosus* M527 to assess transcriptional and translational differences by measuring GUS enzymatic activity. GUS enzymatic activity and total protein content in M527-GA_his_ and M527-NGA_his_ were analyzed and compared. The results showed that over-expression of *nsdA*_*sr*_ led to a significant reduction in protein expression levels. The enzymatic activity of GUS in M527-NGA_his_ (Fig. 12) and its total protein concentration (Table 4) exhibited a significant decline compared to M527-GA_his_, with reductions of 48–56% and 48–53%, respectively. These findings indicate that *nsdA*_*sr*_ over-expression inhibits *rpoB* transcription and subsequently affects overall protein expression, leading to decreased rimocidin biosynthesis.

**Table 4:** The GUS enzyme activity and total protein content of the M527-GA_his_ and M527-NGA_his_

		M527-ES^*^	M527-GA_his_	M527-NGA_his_
GUS enzyme activity (U/g)	48 h	96.73±2.14	93.67±2.33	47.66±1.97
	72 h	94.25±0.89	91.33±1.85	39.57±1.04
Total protein concentration (μg/μL)	48 h	1.24±0.02	1.22±0.02	0.63±0.02
	72 h	0.97±0.02	0.95±0.02	0.48±0.02

^*^M527-ES was obtained in our previous study in which plasmid pGUS-ermE^*^ was integrated into the genome of *S. rimosus* M527 [21]

## Discussion

Rimocidin exhibits a broad-spectrum biological activity [18]. Understanding the molecular regulatory mechanisms governing rimocidin biosynthesis in *S. rimosus* M527 and enhancing its yield represents the long-term objectives of our research. Previous studies have demonstrated that NsdA_sr_ negatively regulates rimocidin biosynthesis [28]. Consequently, elucidating its specific regulatory mechanism is the next logical step in our investigation.

This study initially employed transcriptomic analysis to examine gene expression alterations associated with *nsdA*_*sr*_ over-expression at various time points. The analysis provided comprehensive gene expression data for the M527-NA_sr_ and M527 strains. Comparative analysis revealed that genes associated with central metabolic pathways—including glycolysis, butanoate metabolism, propanoate metabolism, and oxidative phosphorylation—were downregulated in M527-NA_sr_. This downregulation directly impacted the supply of precursors required for rimocidin biosynthesis, a finding further confirmed by intracellular CoA concentration measurements. Moreover, the over-expression of *nsdA*_*his*_ exhibited the negative effects on the cell growth of strain M527, possibly due to the low expression of genes involved in the key metabolic pathway.

Due to the low solubility of the NsdA_sr_ protein, we fused it with a highly specific His-tag to facilitate ChIP-seq experiments. Fermentation results from strains M527-NA_his_, M527-NA, and M527 demonstrated that the expression of NsdA-His did not affect cell growth. Furthermore, fusion with the His-tag had no additional effect on rimocidin production by NsdA_sr_, confirming its suitability for ChIP-seq experiments.

Through bioinformatic analysis of the *S. rimosus* M527 genome (GenBank accession No. GCA_004196335.1) and gene annotations, ChIP-seq results revealed that NsdA_sr_ directly binds to 49 genes, 13 of which have well-defined functions. Notably, one false positive was excluded from the dataset. Among the 12 validated target genes, *RS18275* and *RS18290* are involved in butyrate metabolism, specifically in the synthesis of butyryl-CoA and malonyl-CoA, key precursors for rimocidin biosynthesis. ChIP-seq results for *RS18275* and *RS18290* were further validated through in vitro EMSA experiments, and their downregulation was confirmed by qRT-PCR analysis. Experimental data demonstrated that NsdA_sr_ repressed the expression of these genes, leading to a reduction in intracellular butyryl-CoA and malonyl-CoA levels. This, in turn, resulted in a decrease in rimocidin yield.

Additionally, the *rpoB* gene, which encodes the RNA polymerase β-subunit, was identified as a direct target of NsdA_sr_. NsdA_sr_ binding to *rpoB* resulted in its downregulation. Using *gusA* as a reporter gene to monitor its expression and enzymatic activity in the M527-NA_his_ strain showed a decrease compared to the WT strain. These findings confirm that *nsdA*_*his*_ over-expression directly reduces global protein expression, ultimately leading to decreased rimocidin production.

This study provides new insights into the regulatory role of NsdA_sr_, particularly its influence on precursor availability and protein expression. However, the specific nucleotide sequences to which NsdA_sr_ binds remain unidentified. Determining these sequences could enable the use of gene-editing technologies to mitigate NsdA_sr_’s negative regulatory effects. Furthermore, our study highlights the crucial impact of precursor and cofactor availability on rimocidin biosynthesis. Enhancing the supply of these components may serve as an effective strategy for increasing rimocidin yield. Transcriptomic data also suggest that NsdA_sr_ indirectly influences amino acid metabolism pathways, though the underlying mechanisms require further investigation.

## Conclusion

This study significantly advances our understanding of the regulatory role of NsdA_sr_ through combined transcriptomic and ChIP-seq analyses. Two key findings emerged:

1. NsdA_sr_ directly interacts with genes involved in critical metabolic pathways, thereby reducing the supply of essential precursors and ultimately limiting rimocidin production.
2. NsdA_sr_ modulates global protein expression, further inhibiting rimocidin biosynthesis.

These insights lay the foundation for future research aimed at optimizing rimocidin production through targeted genetic modifications.

## Materials and methods

### Strains, plasmids, primers and culture conditions

All the strains and plasmids used in this study are listed in Table 5. The media and cultural conditions of *Streptomyces* strains and their derivatives, and *Escherichia coli* strains were prepared as described by Yu et al. [12].

**Table 5:** Strains and plasmids used in this study

Strains or plasmids	Description	Source or reference
Strains		
*Escherichia coli* JM109	A general cloning host	Our lab
*Escherichia coli* ET12567 (pUZ8002)	*Cm*^*r*^, *Km*^*r*^, donor strain for conjugation	Our lab
*Escherichia coli* BL21(DE3)	Host strain for protein expression and purification	Our lab
*Streptomyces rimosus* M527	Wild-type strain, rimocidin producer, CCTCC2013270	Our lab
M527-NA_sr_	M527 with integrative vector pIB139-*nsdA*_sr_	Liao et al. [28]
M527-NA_his_	M527 with integrative vector pIB139-*nsdA*_his_	This work
M527-ES	M527 with integrative vector pGUS-ermE^*^	Song et al. [21]
M527-GA_his_	M527 with integrative vector pIB139-*gusA*_his_	This work
M527-NGA_his_	M527 with integrative vector pIB139-*nsdA*-*gusA*_his_	This work
Plasmids		
pIB139	Derivative of integrative plasmid pSET152, harboring a promoter ermE^*^ (PermE^*^), *apr*^r^, *oriT*_*RK2*_, φC31 *int/att*P	Our lab
pIB139-*nsdA*_sr_	Expression of *nsdA*_sr_ gene driven by PermE^*^ in pIB139	Liao et al. [28]
pIB139-*nsdA*_his_	Expression of *nsdA*_his_ gene driven by PermE^*^ in pIB139	This work
pIB139-*gusA*_his_	Expression of *gusA*_his_ gene driven by PermE^*^ in pIB139	This work
pIB139-*nsdA*-*gusA*_his_	DNA fragment harboring PermE^*^ plus *gusA*_his_ inserted in pIB139-*nsdA*_sr_	This work
pGUS-ermE^*^	Expression of *gusA* gene driven by PermE^*^	Our lab
pET28a(+)	Protein expression vector, *Kan*^*r*^	Our lab
pET28a-*nsdA*_sr_	Derived from pET28a, harboring *nsdA*_sr_ gene under the control of T_7_ promoter	This work

All primers with restriction sites (underlined) are listed in Supplementary file: Table S3.

### RNA-Seq analysis

The WT strain *S. rimosus* M527 and the recombinant strain *S. rimosus* M527-NA_sr_ [28] were cultured in shake-flask fermentation for 12, 24 and 36 h. After sampling, they were rapidly frozen in liquid nitrogen and then placed on dry ice and sent to sequencing company for transcriptomic data analysis.

The library construction and RNA sequencing were conducted by Shanghai Majorbio Bio-pharm Technology Co., Ltd. Specifically, the RNA samples underwent treatment to decrease the levels of ribosomal RNA, utilizing the TruSeq^TM^ Stranded Total RNA Library Prep Kit. Subsequently, the RNA transcripts were fragmented through the application of a buffered zinc solution. The cDNA library was constructed through reverse transcription using random hexamers and dUTP-incorporated nucleotides, followed by double-stranded cDNA synthesis and adapter ligation. To achieve strand specificity, the initial cDNA strand was selectively digested with uracil-DNA glycosylase, and the resulting fragments were PCR-amplified before size selection (150–200 bp) *via* denaturing polyacrylamide gel electrophoresis. Final sequencing was performed on the Illumina HiSeq 2000 platform, generating 100-nucleotide single-end reads with standard forward primers.

The sequenced reads were aligned to the *S. rimosus* genome utilizing Bowtie. Subsequently, the read counts for each annotated transcript were meticulously compiled through the application of RSEM, all executed on a web-based platform (cloud.majorbio.com). The software edgeR was used to analyze differential gene expression, and significantly differentially expressed genes were identified based on the following criteria: FDR < 0.05 and|log_2_FC| >= 1.

### Over-expression of *nsdA*_*his*_ in *S. rimosus* M527

The recombinant DNA techniques utilized in this study were performed in accordance with the protocols outlined by Sambrook and Russell [29]. The shuttle vector pIB139 [22, 30] serves as an effective genetic tool, maintaining replication capacity in *E. coli* while enabling site-specific chromosomal integration in *Streptomyces* species. In this context, the genomic DNA of *S. rimosus* M527 was employed as a template to amplify the *nsdA*_*sr*_ open reading frame (ORF) with a His tag *via* PCR, using the primers P-NAhis-F/R (Supplementary file: Table S3). Following this, the PCR product was subjected to digestion with the restriction enzymes *Nde* I and *Xba* I and subsequently inserted into the corresponding *Nde* I/*Xba* I sites of pIB139, resulting in the construction of the plasmid pIB139-*nsdA*_*his*_. The accuracy of the inserted DNA fragment was verified through sequencing, which confirmed the absence of any mutations.

Subsequently, the constructed plasmid pIB139-*nsdA*_his_ was introduced and integrated into genome of *S. rimosus* M527 *via* intergeneric conjugation, resulting in the generation of the recombinant strain *S. rimosus* M527-NA_his_. The successful construction of this recombinant strain was verified through apramycin resistance screening and PCR analysis. The PCR product was sequenced by using sequencing primers. Sequencing results confirmed that no mutations were introduced.

### Examination of the rimocidin production

The fermentation of *S. rimosus* M527, M527-NA_sr,_ and M527-NA_his_, as well as the measurement of rimocidin production were carried out according to the methods as previously described [22, 28].

### Western blot analysis

SDS-PAGE and Western blot analysis were performed by using standard techniques [29].

### ChIP-Seq and qPCR analysis

Samples for ChIP-qPCR were prepared using a conventional chromatin immunoprecipitation protocol as previously described [31–33].

### RNA extraction and RT-qPCR

Extraction of RNA, design of primers, and reverse transcription and qRT-PCR assays were performed according to the methods as previously described [22, 34]. Melting curve analysis was performed under standard conditions to confirm PCR product specificity.

### Determination of intracellular malonyl-CoA and butanoyl-CoA assay

Malonyl-CoA/butanoyl-CoA extraction and quantification were performed with ELISA kits following standard procedures [35, 36].

### Over-expression of *gusA*_his_/*nsdA*_sr_-*gusA*_his_ in *S. rimosus* M527

Using plasmid pGUS-*erm*E^*^ as a template, a DNA fragment harboring *gusA* (encoding β-glucuronidase) open reading frame (ORF) with His tag was amplified by using primers P-GAhis-F1/R1 (Supplementary file: Table S3). The PCR product was subsequently digested with *Nde* I and *Not* I. This digested fragment was then inserted into the corresponding *Nde* I /*Not* I sites of the plasmid pIB139, resulting in the successful construction of the recombinant plasmid pIB139-*gusA*_his_. Further sequencing analysis of the inserted DNA fragment confirmed that the gene was intact and free of any mutations. Subsequently, the constructed plasmid pIB139-*gusA*_his_ was introduced and integrated into the genome of *S. rimosus* M527 *via* intergeneric conjugation, resulting in the generation of the recombinant strain M527-GA_his_.

Using pIB139-*gusA*_his_ as the template, the DNA fragment containing permE^*^ promoter and *gusA*_his_ was cloned by using primers P-permE^*^-F and P-GAhis-R2 (Supplementary file: Table S3), and inserted into pIB139-*nsdA* to obtain recombinant plasmid pIB139-*nsdA*-*gusA*_his_. The recombinant strain *S. rimosus* M527-NGA_his_ was successfully generated by integrating the plasmid pIB139-*nsdA*-*gusA*_*his*_ into the genome of *S. rimosus* M527. The successful construction of the recombinant strains was subsequently confirmed through their apramycin resistance.

### Determination of β-glucuronidase (GUS) enzyme activity and total protein concentration in *S. rimosus* M527-GA_his_ /M527-NGA_his_

- Determination of GUS enzyme activity: The sample cell treatment and GUS detection method were performed as described previously [21, 30, 37, 38].
- Determination of total protein concentration: Protein concentration was determined with the Bradford method with bovine serum albumin used as a standard [39].

### Determination of NADPH and NADH concentrations

NADPH and NADH concentrations were measured using an NADP+/NADPH Assay Kit (JONLNBIO, China) and an NAD+/NADH Assay Kit (Beyotime, China), respectively.

### Protein expression and purification

The *nsdA*_*sr*_ gene was successfully amplified from genomic DNA of *S. rimosus* M527 by PCR using primers P-28a-NA-F/R (Supplementary file: Table S3). The *nsdA*_*sr*_ fragment was treated with *Eco*R I/*Hin*d III restriction enzymes and subsequently inserted into the corresponding sites of the pET28a plasmid. The purification and expression of the His-tagged protein were performed as described previously [12, 40].

### EMSA

EMSAs were performed as previously described [40, 41]. DNA fragments were amplified using primers listed in the Supplementary file: Table S3.

### Statistical analysis

The experimental data presented in this study were derived from three independent biological replicates, with results expressed as mean values ± standard deviation (SD). For comparative analysis, statistical significance was evaluated through Student’s *t*-test.

## Electronic supplementary material

Below is the link to the electronic supplementary material.

Supplementary Material 1

## Data Availability

No datasets were generated or analysed during the current study.

## Abbreviations

Act: Actinorhodin
CCTCC: China Center for Type Culture Collection
CDA: Calcium-dependent antibiotic
GUS: β-glucuronidase
EMSA: Electrophoretic mobility shift assays
HPLC: High-performance liquid chromatography
MS: Mannitol soya flour
ORF: Open reading frame
WT: Wide-type
qRT-PCR: Quantitative RT-PCR
DCW: Dry cell weight
