# Supplementary material for: Study on the regulatory mechanism of NsdAsr on rimocidin biosynthesis in Streptomyces rimosus M527
Source: Microb Cell Fact. 2025 Jul 10;24:162. doi: 10.1186/s12934-025-02784-z (PMC12243394; doi:10.1186/s12934-025-02784-z)
Supplement: Supplementary file 1 — Supplementary Material 1 [file 12934_2025_2784_MOESM1_ESM.docx]

**Supplementary file:**

**Fig. S1** Construction of plasmid pIB139-*nsdA_his_*. A 1476-bp *nsdA_sr_* gene fragment with HIS tag was obtained by PCR using corresponding primers and subsequently inserted into the *Nde* I and *Xba* I sites of plasmid pIB139 based on DNA seamless cloning technology, yielding the plasmid pIB139-*nsdA*_his_.


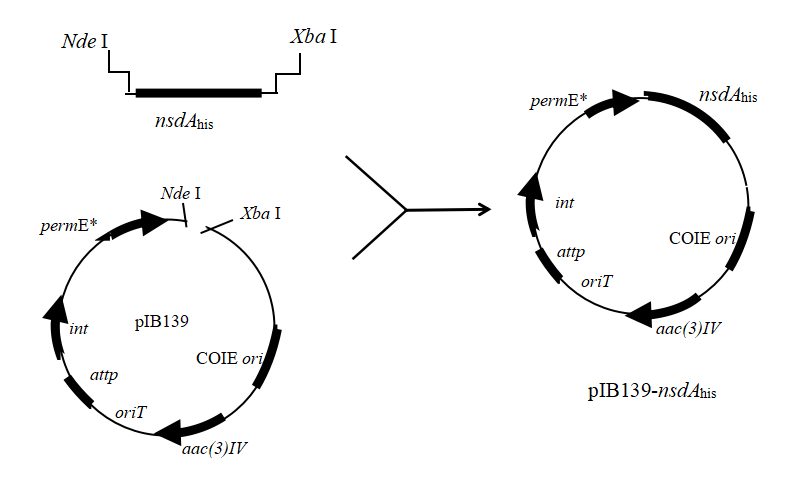


**Fig. S2** Verification of constructed plasmid pIB139-*nsdA_his_*. DNA fragments from the digestion and PCR products were separated on agarose gel. M: DL10000 DNA marker. Lane 1, empty plasmid pIB139 digested with *Nde* I and *Xba* I; lane 2, plasmid pIB139-*nsdA_his_* digested with *Nde* I and *Xba* I; lane 3, PCR product of *nsdA_his_* gene.


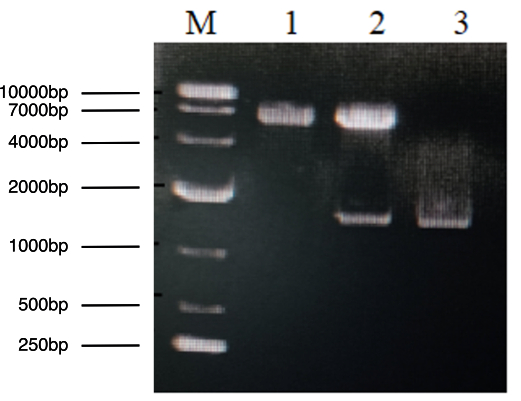


**Fig. S3** Phenotypic verification of recombinant strain *S. rimosus* M527-NA_his_. Recombinant strains *S. rimosus* M527-NA_his_ grew on 2CMC agar medium containing 300 μg/ml apramycin and 100 μg/ml nalidixic acid, while the WT strain *S. rimosus* M527 did not. 2CMC agar medium was incubated at 28 ℃ for 4 days. Number 1-8: eight randomly recombinant strains of *S. rimosus* M527-NA_his_.

_
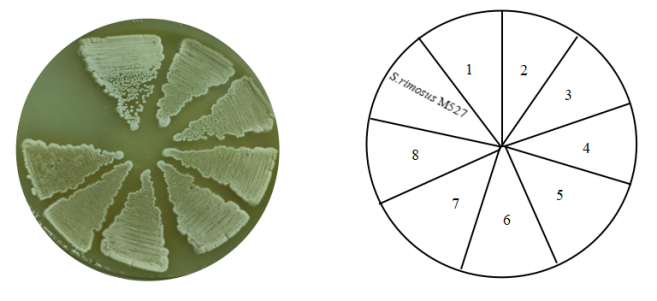
_

**Fig. S4** PCR analysis of apramycin resistance gene *(aac(3)IV*) (a) and DNA fragment harboring *permE*^*^-*nsdA_his_* (b) from initial strain *S. rimosus* M527-NA_his_. DNA fragments from the digestion and PCR products were separated on agarose gel. M: DL2000 DNA marker. Lane 1: PCR product of *aac(3)IV* gene from WT strain *S. rimosus* M527; lane 2: PCR product of *aac(3)IV* gene from plasmid pIB139-*nsdA*_his_; lane 3-7: PCR product of *aac(3)IV* gene from five random strains *S. rimosus* M527-NA_his_(a). Lane 1: PCR product of DNA fragment *permE*^*^-*nsdA_his_* from WT strain *S. rimosus* M527; lane 2: PCR product of DNA fragment *permE*^*^-*nsdA_his_* from plasmid pIB139-*nsdA_his_*; lanes 3-7: PCR product of DNA fragment harboring *permE*^*^-*nsdA_his_* from five randomly selected strains *S. rimosus* M527-NA_his_ by PCR using primers P-*permE*^*^F and P--NAhis-R (b).


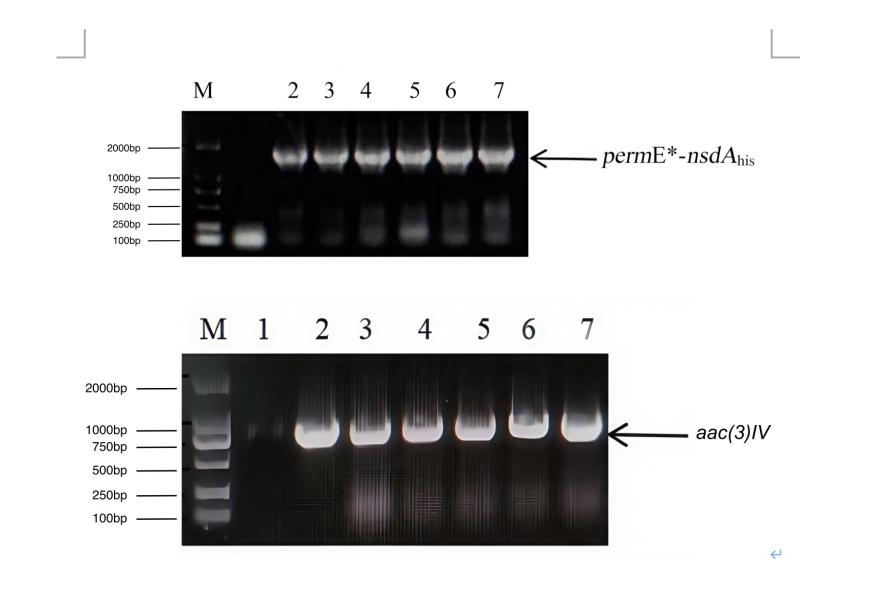


a

**
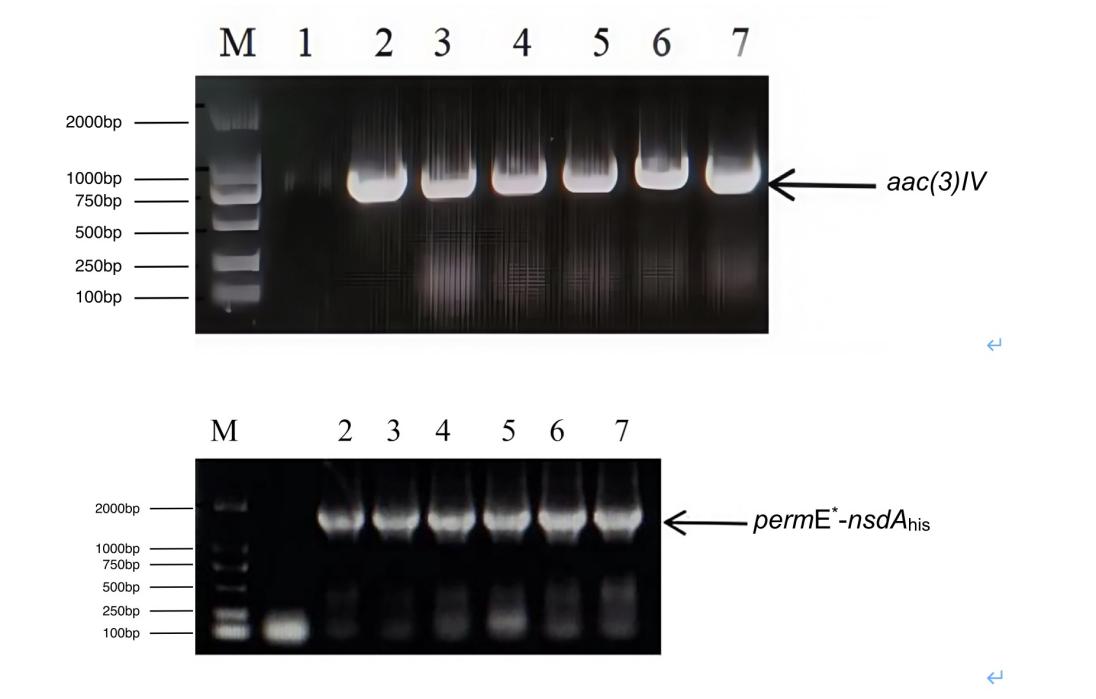
**

b

**Fig. S5** Verification of constructed plasmid pET28a-*nsdA_sr_*. DNA fragments from the digestion and PCR products were separated on agarose gel. M: DL10000 DNA marker. Lane 1, empty plasmid pET28a digested with *Eco*R I and *Hin*d III; lane 2, plasmid pET28a-*nsdA_sr_* digested with *Eco*R I and *Hin*d III; lane 3, PCR product of the *nsdA_sr_* gene.


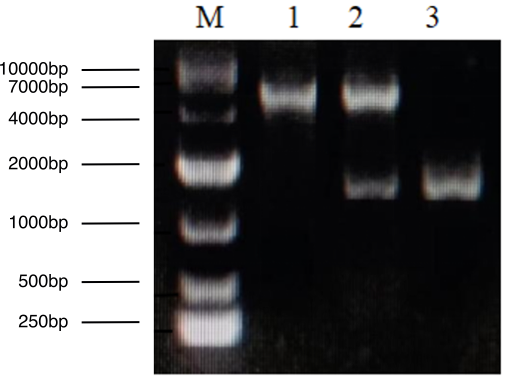


**Fig. S6** IPTG-induced expression and purification of protein NsdA_sr_. M: 130 kDa protein marker. Lane 1, BL21-NsdA_sr_ was not induced to express; Lane 2, BL21-NsdA_sr_ protein was expressed at 37 °C (induced by IPTG for 1 mM); Lane 3, purification of NsdA_sr_ protein.

_
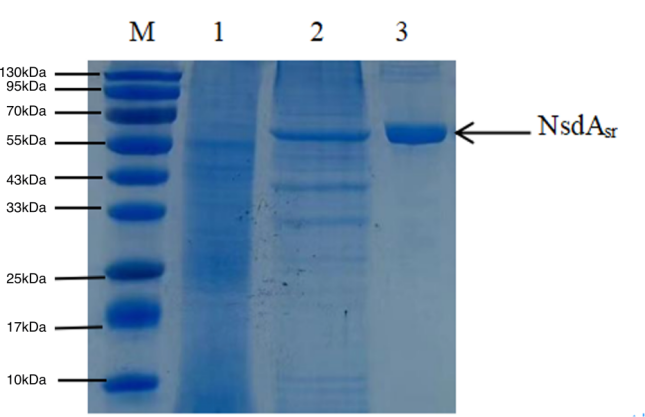
_

**Fig. S7** Construction of plasmid pIB139-*gusA_his_*. A 1812-bp gene *gusA* with HIS tag was obtained by PCR using corresponding primers and inserted into the *Nde* I and *Not* I sites of plasmid pIB139 based on DNA seamless cloning technology, yielding the plasmid pIB139-*gusA_his_*.


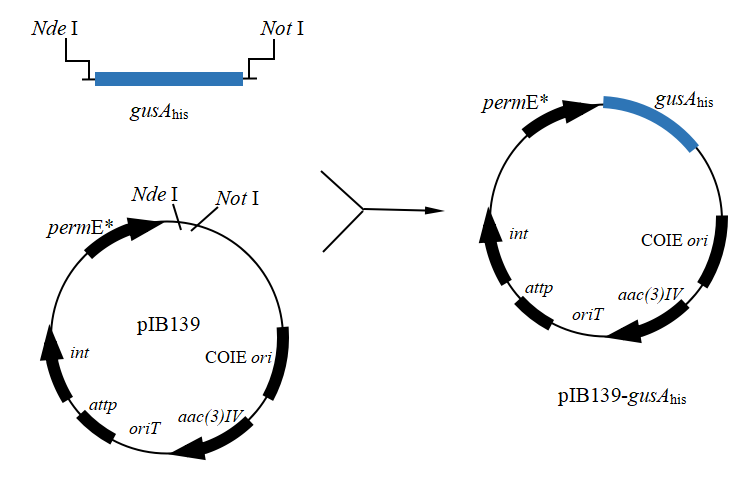


**Fig. S8** Verification of constructed plasmid pIB139-*gusA_his_*. DNA fragments from the digestion and PCR products were separated on agarose gel. M: DL10000 DNA marker. Lane 1, empty plasmid pIB139 digested with *Nde* I and *Not* I; lane 2, plasmid pIB139-*gusA_his_* digested with *Nde* I and *Not* I; lane 3, PCR product of *gusA_his_* gene.


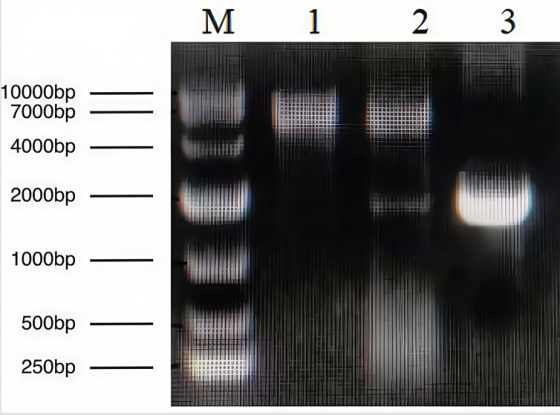


**Fig. S9** Construction of plasmid pIB139-*nsdA*-*gusA_his_*. The fragment *permE*^*^-*gusA_his_* was obtained by PCR using the corresponding primers. A 2041-bp *permE*^*^-*gusA_his_* gene fragment was inserted into the *Eco*R V site of plasmid pIB139-*nsdA* based on DNA seamless cloning technology, yielding the plasmid pIB139-*nsdA*-*gusA_his_*.


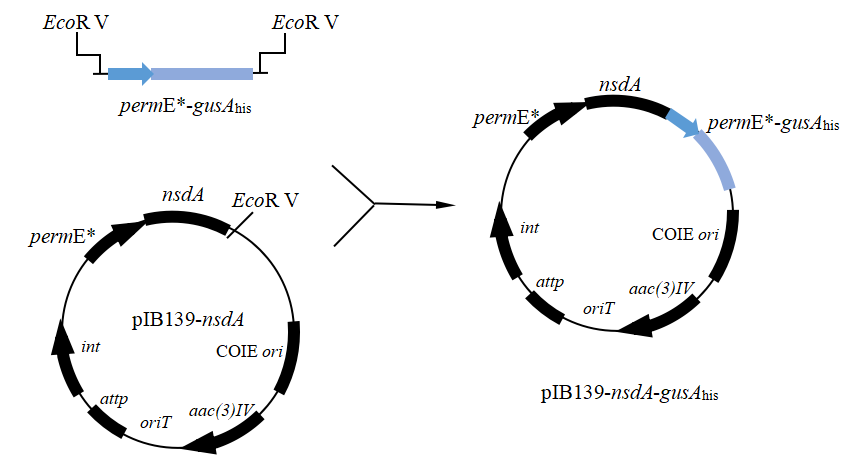


**Fig. S10** Verification of constructed plasmid pIB139-*nsdA*-*gusA_his_*. DNA fragments from the digestion and PCR products were separated on agarose gel. M: DL10000 DNA marker. Lane 1, plasmid pIB139-*nsdA* digested with *Eco*R V; lane 2, plasmid pIB139-*nsdA*-*gusA*_his_ digested with *Eco*R V; lane 3, PCR product of *permE*^*^-*gusA_his_*.

_
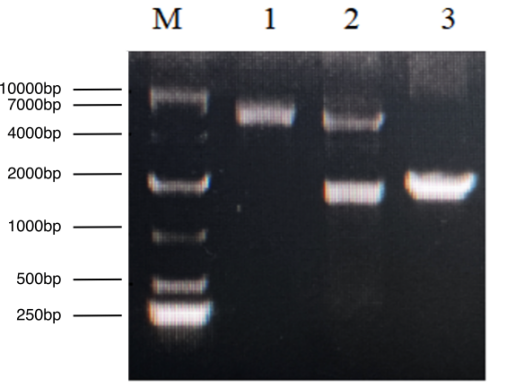
_

**Table S1** Comparison of sequencing data with reference genome^*^

| Sample Name | Total Reads | Genome Mapped Reads | Genome Mapped Ratio(%) | Unmapped Reads | Unmapped Reads Ratio(%) | Uniq Mapped Reads | Uniq Mapped Reads Ratio(%) |
| --- | --- | --- | --- | --- | --- | --- | --- |
| CK_12h | 27093081 | 25227056 | 93.12 | 1866025 | 6.88 | 24410500 | 90.10 |
| CK_24h | 27255604 | 24173202 | 88.68 | 3082402 | 11.32 | 23227958 | 85.21 |
| CK_36h | 26725046 | 23468525 | 87.83 | 3256521 | 12.17 | 22423144 | 83.92 |
| NA_12h | 26569302 | 24156248 | 90.92 | 2413054 | 9.08 | 23662067 | 89.06 |
| NA_24h | 27312322 | 24761231 | 90.66 | 2551091 | 9.34 | 24165198 | 88.48 |
| NA_36h | 27505449 | 24839380 | 90.27 | 2666069 | 9.73 | 24318139 | 88.38 |

^*^ reference genome: *Streptomyces rimosus subsp. rimosus* ATCC 10970 (GCF_000331185.2).

1. Sample Name: wild-type strain *S. rimosus* M527 (CK); *S. rimosus* M527-NA_sr_ (NA);
2. Genome Mapped Reads: the number of reads matched with the reference genome;
3. Genome Mapped Ratio(%): the percentage of reads matched with the reference genome in Clean Reads;
4. Unmapped Reads: the number of reads that are not matched with the reference genome;
5. Unmapped Reads Ratio(%): the percentage of reads that are not matched with the reference genome in Clean Reads;
6. Uniq Mapped Reads: the number of Reads matched to the unique position of the reference genome;
7. Uniq Mapped Reads Ratio(%): the percentage of Reads that match the unique position of the reference genome in Clean Reads.

**Table S2** The top 20 most significant difference terms in KEGG enrichment analysis

| Term | Type II | Type I | DEGs |
| --- | --- | --- | --- |
| Alanine, aspartate and glutamate metabolism | Amino acid metabolism | Metabolism | 44 |
| Nitrogen metabolism | Energy metabolism | Metabolism | 39 |
| Carbon fixation pathways in prokaryotes | Energy metabolism | Metabolism | 38 |
| Pyruvate metabolism | Carbohydrate metabolism | Metabolism | 57 |
| Ribosome | Translation | Genetic Information Processing | 52 |
| Cysteine and methionine metabolism | Amino acid metabolism | Metabolism | 38 |
| Glycine, serine and threonine metabolism | Amino acid metabolism | Metabolism | 53 |
| Glyoxylate and dicarboxylate metabolism | Carbohydrate metabolism | Metabolism | 60 |
| Fatty acid degradation | Lipid metabolism | Metabolism | 43 |
| Oxidative phosphorylation | Energy metabolism | Metabolism | 56 |
| Butanoate metabolism | Carbohydrate metabolism | Metabolism | 50 |
| Amino sugar and nucleotide sugar metabolism | Carbohydrate metabolism | Metabolism | 50 |
| Fatty acid biosynthesis | Lipid metabolism | Metabolism | 42 |
| Quorum sensing | Cellular community - prokaryotes | Cellular Processes | 87 |
| Propanoate metabolism | Carbohydrate metabolism | Metabolism | 39 |
| Purine metabolism | Nucleotide metabolism | Metabolism | 63 |
| Two-component system | Signal transduction | Environmental Information Processing | 95 |
| Glycolysis / Gluconeogenesis | Carbohydrate metabolism | Metabolism | 42 |
| ABC transporters | Membrane transport | Environmental Information Processing | 121 |
| Valine, leucine and isoleucine degradation | Amino acid metabolism | Metabolism | 47 |

**Table S3** Primers used in this study

| Primers | Description (5' to 3') | Source or reference |
| --- | --- | --- |
| P-NAhis-F | ACG*CATATG*CATCATCATCATCATCATGTGGGCGGCAGTGGCGG (*Nde* I) | This work |
| P-NAhis-R | ACG*TCTAGA*TTAGTGGTGGTGGTGGTGGTGGACGGCCTCCGCGCCGG (*Xba* I) | This work |
| P-AP-F | CTCAGACGAGGTGAGGACGACC | Our lab |
| P-AP-R | ACGGACGAGGTGAATCACGAGG | Our lab |
| P-GAhis-F1 | GTTGGTAGGATCCA*CATATG*CATCATCATCATCATCATATGCTGCGGCCCGTCG (*Nde* I) | This work |
| P-GAhis-R1 | GATATCGCGC*GCGGCCGC*TCAGTGGTGGTGGTGGTGGTGCTGCTTCCCGCCCTGCT (*Not* I) | This work |
| P-permE^*^-F | CCGGCGCGGAGGCCGTCTGA*GATATC*TATGCATGCGAGTGTCCGTTCG (*Eco*R V) | This work |
| P-GAhis-R2 | TTACGAATTC*GATATC*TCAGTGGTGGTGGTGGTGGTGCTGCTTCCCGCCCTGCT (*Eco*R V) | This work |
| P-28a-NA-F | TGGGTCGCGGATCC*GAATTC*GTGGGCGGCAGTGGCGGCA (*Eco*R I) | This work |
| P-28a-NA-R | TCGAGTGCGGCCGC*AAGCTT*TCAGACGGCCTCCGCGC (*Hin*d III) | This work |
| YpRS18275-F | GTATCCGCCGTACTGGACTG | This work |
| YpRS18275-R | AAGACCTCGATGAACGTCGC | This work |
| YpRS18290-F | GTTGGACGAGGTGATCGGAG | This work |
| YpRS18290-R | CACAACAGCTTCGCCACAC | This work |
| YpRpoB-F | CTGATCCAGAACCAGGTCCG | This work |
| YpRpoB-R | GGTGCCGAAGAACTCCTTGA | This work |
| PrpoB-Biotin or Wild-F | GAGAAGAACAAAGAGGGGTACC | This work |
| PrpoB-Biotin or Wild-R | AACACAGCCGGAGGAAAG | This work |
| RS18275-Biotin or Wild-F | GAGAAACCCGTCCTCGCC | This work |
| RS18275-Biotin or Wild-R | ACGACTCGGTTGACCAGG | This work |
| RS18290-Biotin or Wild-F | TTGGGCGGGAGTTGGACG | This work |
| RS18290-Biotin or Wild-R | CCCAACCGCTGATGCCAC | This work |
